# Effect of Several New and Currently Available Oxime Cholinesterase Reactivators on Tabun-intoxicated Rats

**DOI:** 10.3390/ijms9112243

**Published:** 2008-11-14

**Authors:** Jana Zdarova Karasova, Jiri Kassa, Young-Sik Jung, Kamil Musilek, Miroslav Pohanka, Kamil Kuca

**Affiliations:** 1 Department of Toxicology, Faculty of Military Health Sciences, Trebesska 1575, 500 01 Hradec Kralove, Czech Republic. E-Mails: kassa@pmfhk.cz (J. K.); kucakam@pmfhk.cz (K. K.); 2 Center of Advanced Studies, Faculty of Military Health Sciences, Hradec Kralove, Czech Republic; 3 Medicinal Science Division, Korea Research Institute of Chemical Technology, PO Box 107, Yusong, Taejon 305-606, Korea

**Keywords:** Acetylcholinesterase, butyrylcholinesterase, reactivators, oximes, pretreatment

## Abstract

The therapeutical efficacies of eleven oxime-based acetylcholinesterase reactivators were compared in an *in vivo* (rat model) study of treatment of intoxication caused by tabun. In this group there were some currently available oximes (obidoxime, trimedoxime and HI-6) and the rest were newly synthesized compounds. The best reactivation efficacy for acetylcholinesterase in blood (expressed as percent of reactivation) among the currently available oximes was observed after administration of trimedoxime (16%) and of the newly synthesized K127 (22432) (25%). The reactivation of butyrylcholinesterase in plasma was also studied; the best reactivators were trimedoxime, K117 (22435), and K127 (22432), with overall reactivation efficacies of approximately 30%. Partial protection of brain ChE against tabun inhibition was observed after administration of trimedoxime (acetylcholinesterase 20%; butyrylcholinesterase 30%) and obidoxime (acetylcholinesterase 12%; butyrylcholinesterase 16%).

## 1. Introduction

Organic compounds based on phosphorus exert a broad variety of biological properties. From toxicological point, especially when biological effect is taken into consideration, organophosphorus inhibitors (OPI) of cholinesterases (ChE) are the most important chemicals of this group [1]. The inhibitory effect is based on phosphorylation or phosphonylation of the serine hydroxyl group at the ester part of the ChE active site of the enzyme [2].

The standard treatment for OPI intoxication usually consists of administration of anticholinergic drugs (e.g. atropine), in combination with reactivator containing an oxime functional group in its structure. Anticholinergic drugs block the effects of overstimulation by accumulated acetylcholine at peripheral muscarinic receptors while the oximes, as compounds with nucleophilic activity, repair the enzyme by dephosphorylation and restore ChE activity [3]. However, the efficacy of commonly used oxime reactivators is still not sufficient.

**Figure 1.**
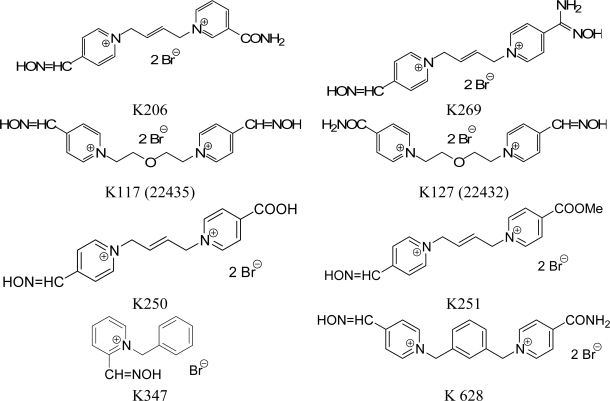
Chemical structures of newly synthesized oximes.

Tabun (GA agent; *O*-ethyl-*N,N*-dimethylphosphoramido cyanide) belongs to the nerve agents that could be misused for military purposes. Commonly used bisquaternaly reactivators of acetylcholinesterase (AChE; EC 3.1.1.7) are not able to counteract the toxic effects of tabun because of their very low reactivating efficacy [4, 5], which has been explained by difficulties with the nucleophilic attack on the tabun phosphorus atom [5, 6]. The development of new and more effective AChE reactivator is still a challenge. For these reasons, eight new reactivators (Figure 1) of tabun-inhibited ChE have been synthesized [7–12] in an attempt to increase the reactivating efficacy of antidotes for treatment of poisoning by this nerve agent. These compounds were, according to our *in vitro* results, very promising against tabun [9–13].

**Figure 2.**
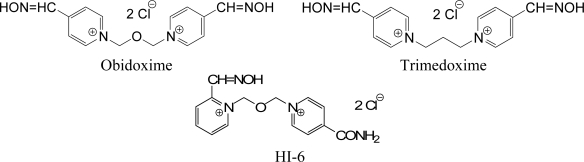
Chemical structures of currently available oximes.

The aim of this study was *in vivo* evaluation of reactivating effects of currently available oximes (HI-6, obidoxime and trimedoxime) (Figure 2) and the eight newly synthesized oximes (K206, K269, K117 - 22435, K127 - 22432, K250, K251, K347, K628) in combination with atropine (a commonly used anticholinergic drug) in tabun-poisoned rats. Syntheses as well as analyses of these reactivators were previously published [7–13]. The other aim of this study was to compare the reactivation potency of these compounds against tabun-inhibited butyrylcholinesterase (BChE; EC 3.1.1.8). These *in vivo* data could be useful for preparation of effective pretreatment therapy including administration of pseudo-catalytic bioscavenger.

## 2. Results and Discussion

**Figure 3.**
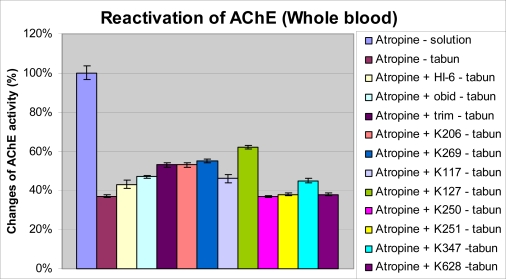
Changes of AChE activity in whole blood after tabun intoxication and administration of AChE reactivators.

Tabun intoxication produces a strong depression of AChE activity in blood to approximately one third of the original activity. Among the currently available oximes, trimedoxime was found to be the best reactivator of the tabun-inhibited AChE. Among the newly synthesized oximes, the best result was obtained after administration of K127 (22432). Some oximes were irresponsive to tabun-inhibited AChE (K250, K251 and K628). The therapeutical effects of K206 and K269 were comparable with effect of trimedoxime. Results are summarized in Figure 3.

**Figure 4.**
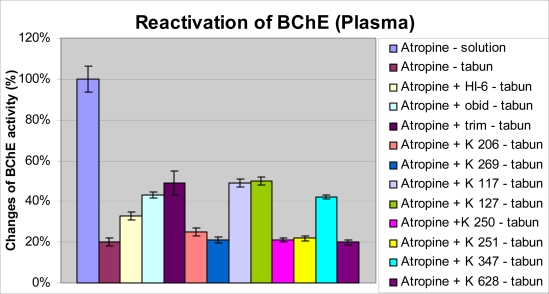
Changes of BChE activity in plasma after tabun intoxication and administration of oxime reactivators.

The BChE activity was strongly decreased after tabun intoxication (Figure 4). The residual activity of tabun-inhibited BChE was 20 % of the original activity. Among the currently available oximes, trimedoxime was again the best. Some of newly synthesized oximes were not able to significantly reactivate tabun-inhibited BChE (K269, K250, K251, K628) and some (K117 - 22435, K127 - 22432) were as good as trimedoxime.

**Figure 5.**
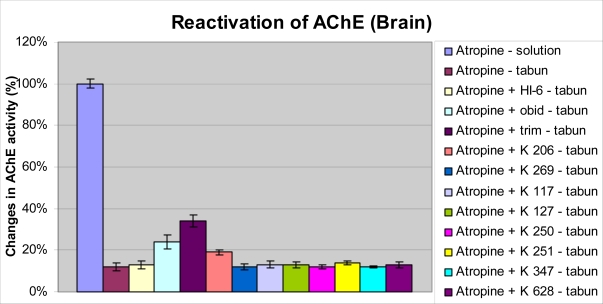
Changes of AChE activity in brain after tabun intoxication and administration of oxime reactivators.

**Figure 6.**
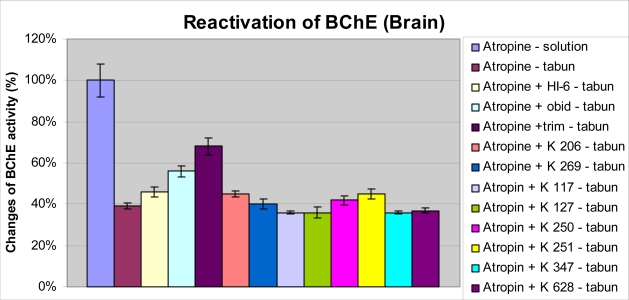
Changes of BChE activity in brain after tabun intoxication and administration of oxime reactivators.

Strong inhibition of both cholinesterases was recorded in central nervous system (CNS) (Figures 5 and 6). The inhibition of BChE was not so strong with respect to peripheral compartment (plasma). Only trimedoxime and obidoxime from the reactivators were able to protect part of cholinesterases in brain tissues. Trimedoxime seems to be the best reactivator again, because it was able to increase AChE and BChE activity by more than 20% in CNS.

Generally, the efficacy of ChE reactivators depends on their reactivity and affinity towards organophosphate-inhibited enzymes. Their reactivity is derived from the nucleophilic activity of the oxime anion that is bound on a pyridinium ring [14]. They differ from each other by the position of the oxime group on the pyridinium ring and the linker between pyridinium rings. The affinity of oximes for intact enzyme is determined by various physicochemical factors such as electrostatic attraction and repulsion, hydrophobic interactions and by the shape and size of the whole molecule as well as the functional groups [15].

Bisquaternary reactivators have higher affinity towards both intact and inhibited ChE and higher potency to reactivate nerve agent-inhibited ChE compared to monoquaternary ones [16]. One of the used reactivators, oxime K347, is a monoquaternary oxime with a benzyl group at the one position on the pyridinium ring, the reactivating potency of this compound was better than that of some bisquaternary oximes.

The position of the oxime group on the pyridinium ring is another factor influencing the reactivation efficacy. The oxime reactivators with the oxime group in the position 4 are the best reactivators of tabun-inhibited AChE [17]. The reactivators used in this comparative study, except for oxime K347 and HI-6, all had the oxime group in this position.

The linking chain between two pyridinium rings is another important factor influencing the potency of oximes. Although this part of the oxime reactivator molecule does not play any role in the dephosphorylation process, it is a major factor influencing reactivation rates [18,19]. There is also only one difference between the composition of the obidoxime and trimedoxime connection chains (linkers). In several cases (oximes K206, K269, K250, K251), linkers with rigid double bond were used. Although K269 and K206 have good reactivating potency against tabun-inhibited AChE, if compared with the current best oxime against tabun - trimedoxime, in case of BuChE, their effect is not sufficient. Oximes K117 (22435) and K127 (22432) have longer linking chains with oxygen atoms in their structure. Their reactivation efficacies were different from those of other tested oximes. Oxime K127 (22432) was better than the currently used trimedoxime. Oxime K117 (22435) was comparable to obidoxime. Surprisingly, these oximes had good reactivating potency against tabun-inhibited BChE.

Carboxyl and methylcarbonyl groups on one of pyridinium rings (K250, K251) decreased the reactivation potency of tested oximes in case of both ChEs. The other important factor influencing oxime efficacy is its pharmacokinetics, as its distribution from the muscle to the blood stream plays an important role. The ability of oxime reactivators to go through the blood-brain barrier in an effective level and subsequently to have reactivation effect in brain is a hotly discussed topic nowadays [20–23].

The inhibitions of ChE in CNS after administration of tabun were strong. The depression of AChE was stronger than inhibition of BChE. The reactivations of both ChE using newly synthesized oximes were not statistically significant. Only trimedoxime and obidoxime were able to protect the ChE in brain. This may be caused by better penetration through blood-brain barrier of these oximes.

## 3. Experimental Section

### 3.1. Chemicals

The nerve agent tabun (*O*-ethyl-*N,N*-dimethylphosphoramido cyanide) of 98% purity was obtained from the Military Technical Institute of Protection (Brno, Czech Republic) and stored in glass ampulles (0.3 mL). For the experiments solutions were prepared immediately before use due to its spontaneous hydrolysis during the long term storage [24]. All other chemicals were obtained from Sigma-Aldrich (Prague, Czech Republic). Oximes were synthesized at the Department of Toxicology, Faculty of Military Health Sciences (Hradec Kralove, Czech Republic) [8–11].

### 3.2. Animals

Male Wistar rats, weighing from 180 to 200 g, were purchased from Anlab s.r.o. (Prague, Czech Republic). The animals were maintained in an air-conditioned room (the temperature were 22 ± 2 ^°^C, the humidity was 50 ± 10%, with light from 7 a.m. to 7 p.m.), and were allowed free access to standard chow and tap water.

Housing of animals was realized in the Central Vivarium of the Faculty of Charles University and Faculty of Military Health Sciences, Hradec Kralove.The experiment was performed under permission and supervision of the Ethic Committee of the Medical Faculty of Charles University and Faculty of Military Health Sciences, Hradec Kralove.

### 3.3. Intoxication and sample collection

Although the main route of administration of organophosphorus nerve agents is percutaneous or inhalation, we used intramuscular administration (i.m.) in our experiments, because there is better comparability with already available data [25]. A single dose of 1 × LD_50_ of tabun was injected i.m.. Before each experiment, orientation toxicity determination was done to prove that the dose to be administered really corresponds to the single LD_50_.dose.

Oximes in therapeutical dosages (5 % LD_50_) in combination with therapeutical dose of atropine (21 mg/kg) were administered i.m. 5 min before intoxication. First control group was treated only by therapeutical dose of atropine and after 5 min was injected saline solution i.m. instead of nerve agent. The second control group of animals was treated also by therapeutical dose of atropine and after 5 min tabun was administered.

Rats were anesthetized with CO_2_ and killed by decapitation 30 min after the nerve agent intoxication. After decapitation, the trunk blood was collected in heparinized tubes and one part of this blood separated into plasma and erythrocytes by centrifugation (3000 × g for 15 min, 15^°^C) with Universal 320R (Hettich, Germany). The brains were removed from the sculls and stored at –80^°^C until the assay.

### 3.4. Biochemical examinations

The whole blood was measured just in day, when the samples were collected. The blood samples were hemolyzed by using 0.02M Tris buffer, pH 7.6 (ratios: 1:20) for 5 min. Plasma samples were stored as other tissues until the assay. After thawing, brains were homogenized (weight of tissue: volume - 1:10; 0.02M Tris buffer, pH 7.6). Each sample was mixed with Ultra-Turrax homogenizer (Janke & Kunkel, Germany) for 20 seconds. These homogenates were used for enzymatic analysis.

The activities of AChE and BChE were assessed by standard spectrofotometric Ellman’s method with acetylthiocholine or butyrylthiocholine iodides as substrates and 5,5‘-dithiobis(2-nitrobenzoic) acid as a chromogen [26], modified in wavelength 436 nm (because of influence caused by hemoglobin). The spectrophotometer Helios Alpha (Electron Corporation, Great Britain) was used for determination of absorbancy. The results were expressed as μcat/g of wet weight tissue.

### 3.5. Statistical evaluation

The percent of reactivation R was taken as an outputting parameter. The value of the R was calculated according following equation:

$$R=\frac{\Delta A_{r}-\Delta Ai}{\Delta A_{0}-\Delta Ai}\times 100(\%)$$

The symbol A_0_ means absorbance provided by mixture with intact AChE (in this mixture was no inhibitor as well as no reactivator), A_i_ is absorbance of mixture with inhibited AChE (no reactivator) and A_r_ indicates AChE activity influenced by inhibitor and also reactivator [27].

The number of animals per group was 6. Enzyme activities in tissue homogenates were expressed as the mean ± standard deviation (n=6) and statistical difference were tested by *t*-test using Graph Pad.

## 4. Conclusions

In conclusion, the majority of the newly synthesized oximes were unable to surpass the efficacy of a currently used drug – obidoxime. Only trimedoxime in both compartments and K127 (22432) in peripheral tissue were partially effective. Some newly synthesized oximes (K117 - 22435; K347) were as effective as oxime HI-6, but at levels insufficient for treatment of tabun-poisoning. No reactivation effect was found after administration of K628, K250, and K251. The partially protection of brain ChE against tabun was found only after administration of trimedoxime and obidoxime. However, these arguments might not hold in the case of soman poisoning [28].
